# H3K4 demethylase SsJMJ11 negatively regulates drought-tolerance responses in sugarcane

**DOI:** 10.1186/s12870-025-06832-z

**Published:** 2025-07-02

**Authors:** Guangrun Yu, Xiaoge Wu, Meiling Ye, Yuan Fang, Qiongli Wang

**Affiliations:** 1 Key Laboratory of Ministry of Education for Genetics, Breeding and Multiple Utilization of Crops, College of Agriculture, Fujian Agriculture and Forestry University, Fuzhou, Fujian 350002 China; 2 State Key Laboratory of Tropical Crop Breeding, Shenzhen Branch, Guangdong Laboratory for Lingnan Modern Agriculture, Genome Analysis Laboratory of the Ministry of Agriculture and Rural Affairs, Agricultural Genomics Institute at Shenzhen, Chinese Academy of Agricultural Sciences, Shenzhen, China

**Keywords:** Drought stress, Histone demethylase, JmjC protein, H3K4me3, *Saccharum spontaneum*

## Abstract

**Background:**

Drought-induced gene alteration is usually associated with changes of histone H3K4me3 in plants. Histone methylation homeostasis relies on the coordinated activity of methyltransferases and demethylases. We previously demonstrated that SsJMJ11 is an H3K4me3 demethylase in *Saccharum spontaneum* and participates in regulating flowering time. However, the role of H3K4me3 regulators in regulating drought-stress responses in sugarcane (*Saccharum* spp.) remains elusive.

**Results:**

We show that SsJMJ11 negatively regulates drought-stress responses by acting as an H3K4me3 demethylase. Ectopic overexpression of *SsJMJ11* in *Arabidopsis thaliana* resulted in a hypersensitivity to soil drought stress as well as abscisic acid (ABA) and mannitol. Meanwhile, the drought-induced expression of *AtRD20* and *AtDREB2A*, two well-known positive regulators of drought tolerance, was repressed by SsJMJ11 overexpression. In *S. spontaneum*, the ABA- and dehydration-induced transcription of *SsRD20* and *SsDREB2A* was associated with increased H3K4me3 levels at these loci. Furthermore, transient overexpression of *SsJMJ11* in *S. spontaneum* protoplasts reduced the ABA-induced transcription of *SsRD20* and *SsDREB2A*, paralleling reduced H3K4me3 levels at these loci.

**Conclusions:**

Our results suggest that SsJMJ11-mediated dynamic deposition of H3K4me3 is required for proper adaptation to drought stress in sugarcane.

**Supplementary Information:**

The online version contains supplementary material available at 10.1186/s12870-025-06832-z.

## Introduction

Sugarcane is a vital economic crop producing 80% of the global sugar and 24% of the global ethanol [1]. Currently, conventional sugarcane breeding faces significant challenges in adapting to the increasing global demand and unfavorable environmental conditions. Drought has emerged as a leading environmental stress limiting sugarcane production, and drought adaptation is a key trait for sugarcane breeding [2]. Thus, it is urgent to clarify the mechanisms underlying sugarcane drought responses and to develop drought-tolerant sugarcane varieties. However, the large and complex polyploid genome of sugarcane, its recalcitrance to genetic transformation, and long breeding cycle have posed a challenge for the development of drought-tolerant varieties [3]. The recent breakthroughs in the modern sugarcane genome research and newly developed biotechnological approaches provide an opportunity to functionally characterize drought-resistance genes in sugarcane [1, 4].

As sessile organisms, plants have developed complex protective strategies during their long evolutionary history to adapt to various adverse environmental conditions [5, 6]. When exposed to drought stress, the expression of drought-responsive genes is associated with substantial changes in histone modification [7, 8]. Histone H3 lysine 4 trimethylation (H3K4me3) is an active marker predominantly located near the transcription start sites (TSS) of actively transcribed genes [9]. It has been reported that H3K4me3 levels increase significantly at drought-induced genes, including *AtRD20* and *AtRD29A* under drought conditions, while the modification gradually decreases, coinciding with downregulation of these drought-responsive genes’ expression during rehydration [10]. In addition, H3K4me3 participates in the modulation of drought-stress memory. H3K4me3 persists at some dehydration-responsive gene loci after rewatering, which allows faster and stronger transcriptional activation of these stress response genes in subsequent drought stresses [10].

The homeostasis of histone methylation is dynamically regulated by histone methyltransferases and demethylases [11, 12]. Jumonji C (JmjC) domain-containing proteins are conserved histone demethylases that remove methyl groups from methylated histones at lysine residues [12, 13]. JmjC domain-containing histone demethylases are categorized into five distinct subfamilies based on their catalytic domain sequence in plants, including the KDM3/JHDM2 group, KDM4/JHDM3 group, KDM5/JARID1 group, JMJD6 group, and the JmjC domain-only group [14, 15]. Among the JmjC proteins, Arabidopsis *AtJMJ14*, *AtJMJ15*,* AtJMJ16*,* AtJMJ17*,* AtJMJ18*,* AtJMJ19* and *Oryza sativa OsJMJ703*,* OsJMJ704* are identified as members of the KDM5/JARID1 group, which actively demethylates H3K4me1, H3K4me2, and H3K4me3 [16–18]. Evidence has revealed that H3K4 demethylases play critical roles in regulating diverse biological processes, such as gene silencing, flowering, and senescence [19–24]. In addition, H3K4 demethylases are also involved in drought-stress response regulation. In Arabidopsis, the *jmj17* mutant displayed dehydration stress tolerance and ABA hypersensitivity. AtJMJ17 binds directly to the chromatin of *OPEN STOMATA 1* (*AtOST1*) and remove H3K4me3 for the regulation of *AtOST1* expression, thereby modulating the dehydration stress response [25]. The H3K9 demethylase AtJMJ27 positively regulates drought-stress responses by promoting the expression of *AtGOLS2* and *AtRD20* [26]. In rice, H3K4me3 levels at dehydration-responsive gene loci were significantly increased in the *jmj703* mutant. Overexpression of *OsJMJ703* leads to sensitivity to drought stress, whereas knockdown of *OsJMJ703* enhances drought-stress tolerance [27]. These findings suggest that the dynamic removal of H3K4 methylation on histones mediated by JmjC proteins is essential for ensuring proper adaptation to drought stress. However, it remains unclear how drought stress modulates the H3K4me3 levels of drought-responsive genes in sugarcane.

The *S. spontaneum* genome encodes 26 JmjC domain-containing histone demethylases (SsJMJ1-SsJMJ26) [28]. Our recent study revealed that KDM5/JHDM1 subfamily JmjC protein SsJMJ11 is an H3K4me1/2/3 demethylase involved in promoting flowering in *S. spontaneum*, while SsJMJ26 is a specific H3K4me3 demethylase [29]. In this study, we found that SsJMJ11 negatively regulates drought-stress responses by acting as an H3K4me3 demethylase. Our data indicated that SsJMJ11 represses the expression of drought-induced *SsRD20* and *SsDREB2A*, which is associated with reduced H3K4me3 levels at these loci under drought stress. Our findings reveal a novel function of *SsJMJ11* in modulating drought-stress responses.

## Methods

### Plant materials and growth conditions

The *S. spontaneum* accession (SES208) used in this study was provided by the Center for Genomics and Biotechnology, Fujian Agriculture and Forestry University, Fuzhou, China. The Col-0 ecotype of *A.thaliana* was used for transformation to generate transgenic lines. Sugarcane seedlings were cultivated under controlled conditions in a growth chamber simulating natural environments, with a 16-h light/8-h dark photoperiod, 60% relative humidity, and a constant temperature of 28 °C [30].

### Stress treatment

For drought stress treatment in *S. spontaneum*, six-week-old seedlings were exposed to controlled drought conditions. Relative soil water content (RSWC) was closely monitored, and samples were collected at RSWC levels of 70% (control) and 10% (drought) for comparative analysis. For ABA, methyl jasmonate (MeJA), and abiotic stress treatments, four-week-old hydroponically grown *S. spontaneum* seedlings were treated with 100 µM ABA, 200 mM NaCl (salt stress), 100 mM mannitol, 30% PEG6000, 100 µM MeJA, 4 °C (cold stress), and 38 °C (heat stress) for 0, 3, 6, and 12 h.

For dehydration treatment, leaves of four-week-old *S. spontaneum* and three-week-old Arabidopsis were dehydrated for 0, 1, and 2 h. After treatment, the seedlings were rapidly frozen and stored at -80 °C for molecular analyses. Each treatment was replicated three times, with each replicate consisting of nine seedlings, ensuring the reliability and reproducibility of the experimental data.

### Drought tolerance assessment

We evaluated the response of transgenic Arabidopsis to drought stress under soil conditions following the standard methods described by Wang et al. [26]. Seedlings were grown in a controlled environment at 22 °C and a 16-h light/8-h dark cycle for about two weeks, and the seedlings were subjected to drought treatment until the RSWC dropped to approximately 5% and then rehydrated for resuming. Three days after rewatering, each plant was photographed, and survival rates were recorded.

### Plasmid construction and generation of transgenic Arabidopsis plants

The primer sequences for constructing the plasmids *SUPERpro: SsJMJ11-GFP* and *SUPERpro: SsJMJ26-GFP* were as follows: SsJMJ11-Hind III-Forward 5′-aaatcgactctagaaagcttATGCTTTCCACATCCGCCGAGG-3′, SsJMJ11-KpnI-Reverse 5′ -tgctcaccatggtaccGGTGGTCCTGTGATCCTGTCCC-3′, SsJMJ26-Hind III-Forward 5′-aaatcgactctagaaagcttATGCTTGCAATGATGGGAACAG-3′, SsJMJ26-KpnI-Reverse 5′ -tgctcaccatggtaccGTGCCATTGCTTTGCAATCTCC-3′. Using these primers and high-fidelity DNA polymerase, we performed PCR amplification to obtain accurate fragments of the *SsJMJ11* and *SsJMJ26* genes. Subsequently, the gene fragments were inserted into the *pSUPERpro1300:GFP* vector, which had been linearized with HindIII and KpnI, via homologous recombination. After sequence verification, the recombinant plasmids were introduced into *Agrobacterium tumefaciens* GV3101 competent cells. Thereafter, *Agrobacterium* harboring the target genes was transferred into wild-type *A. thaliana* (Col-0) plants using the floral dip method. Following stringent selection, homozygous T3 generation transgenic *A. thaliana* plants stably expressing the SsJMJ11-GFP or SsJMJ26-GFP fusion proteins were generated. These transgenic plants exhibited stable fluorescence signals and served as the basis for subsequent functional analyses and phenotypic observations. All primer sequences used for constructing the recombinant plasmids are provided in Supplementary Table 1.

### In vitro leaf water loss, stomatal development and movement analysis

For leaf water loss, rosette leaves from four-week-old plants were excised and placed in a laboratory environment maintained at 22 °C to measure water loss. High-precision electronic scales were used to record the weight changes of the rosette leaves every hour for 10 h. The water loss rate (%) was calculated using the formula: (initial fresh weight - final fresh weight) / initial fresh weight × 100%.

For stomatal movement analysis, we analyzed the apertures of stomata as described previously [26]. Rosette leaves from four-week-old plants were incubated in a stomata-opening solution (10 mM MES, pH 6.15; 50 mM CaCl₂; 10 mM KCl) at 22 °C for 3 h to promote stomatal opening, and followed by a 3-hour incubation in a solution containing 5 µM ABA and 5 µM CaCl₂ to induce stomatal closure. Subsequently, epidermal tissues were carefully peeled and mounted on slides. Stomatal apertures were imaged using an Olympus BX63 microscope, and the degree of stomatal opening and closing was quantitatively analyzed with ImageJ software (National Institutes of Health, USA).

For stomatal density analysis, equally sized epidermal samples were taken from the distal third of the leaves. The epidermal layers were imaged using an Olympus BX63 microscope, and the stomatal density (number of stomata per square millimeter) was calculated. Additionally, the stomatal index, defined as the ratio of the number of stomata to the total number of epidermal cells within the field of view, was determined. This serves as a supplementary assessment metric for stomatal distribution characteristics [31].

### Real-time quantitative polymerase chain reaction (RT-qPCR) analysis

Total RNA was extracted from *S. spontaneum* seedlings, rosette leaves of three-week-old Arabidopsis plants with various treatments and *S. spontaneum* protoplasts with GFP or SsJMJ11-GFP transformed following the Omega R6827 kit manual. For RT-qPCR assays in *S. spontaneum* protoplasts, transformation of *S. spontaneum* protoplasts was performed as the protocol described in the previous studies [28, 29]. Protoplasts transformed with GFP or SsJMJ11-GFP were used as starting materials after adjustment with un-transformed protoplasts to ensure that the number of transformed protoplasts in the two groups is approximately the same. The RNA was reverse-transcribed into cDNA using the Genstar A224-10 kit. RT-qPCR was performed using SYBR Premix ExTaq on an Applied Biosystems StepOnePlus system. *A. thaliana AtUBC/AtACTIN2* and *S. spontaneum SseEF/SsACTIN2* were selected as reference genes. All experiments were independently repeated at least three times. Consistent results were obtained with different reference genes; thus, only the results using *AtUBC* and *SseEF* are presented. The primers used in this experiment are provided in Supplementary Table 1.

### ChIP-qPCR analysis

The ChIP experiment was carried out following the method described as previously [26]. *S. spontaneum* seedlings and protoplasts transformed with GFP and SsJMJ11-GFP after adjustment were used as starting materials. Immunoprecipitation of H3K4me3 histone was accomplished using ProteinA Dynabeads (Invitrogen #10001D) coupled with H3K4me3 antibody (Millipore 07-473); immunoprecipitation of SsJMJ11-GFP was achieved using a GFP antibody (Abcam ab290). Quantitative PCR was employed to analyze the purified DNA fragments. ChIP signals were normalized relative to input DNA, and each experiment was replicated at least three times biologically. Primer sequences are provided in Supplementary Table 1.

### Orthogroup analysis

Using the amino acid sequences of *AtRD20* and *AtDREB2A* from Arabidopsis as templates, we performed a BLASTP search against the *S. spontaneum* genome to identify potential candidate sequences. Subsequently, we conducted a detailed analysis of these candidate sequences using OrthoFinder v2.2.7 [32] with default settings to elucidate their homology. Finally, we completed a multiple sequence alignment of the aforementioned homologous amino acid sequences using DNAMAN software, providing a foundation for subsequent functional analyses.

### Accession numbers

Sequence data from this study can be found in the *S. spontaneum* genome database (http://www.life.illinois.edu/ming/downloads/Spontaneum_genome/) and Arabidopsis genome initiative database under the following accession numbers: *SsJMJ11* (Sspon.02G0043430-1B), *SsJMJ26* (Sspon.08G0010180-2B), *SsRD20* (Sspon.05G0007230-1 A), *SsDREB2A* (Sspon.03G0036110-1B), *SsACTIN2* (Sspon.05G0018830-2B), *SseEF* (Sspon.02G0008140), *AtRD20* (AT2G33380), *AtDREB2A* (AT5G05410), *AtABI5* (AT2G36270), *AtOST1* (AT4G33950), *AtABF1* (AT1G49720), *AtABF2* (AT1G45249), *AtABF3* (AT4G34000), *AtABF4* (AT3G19290), *AtGOLS2* (AT1G56600), *AtERD1* (AT5G51070), *AtERD10* (AT1G20450), *AtERD11* (AT1G02930), *AtHB7* (AT2G46680), *AtACTIN2* (AT3G18780) and *AtUBC* (AT5G25760).

## Results

### *SsJMJ11* and *SsJMJ26* are responsive to drought stress in *S. spontaneum*

**Fig. 1:**
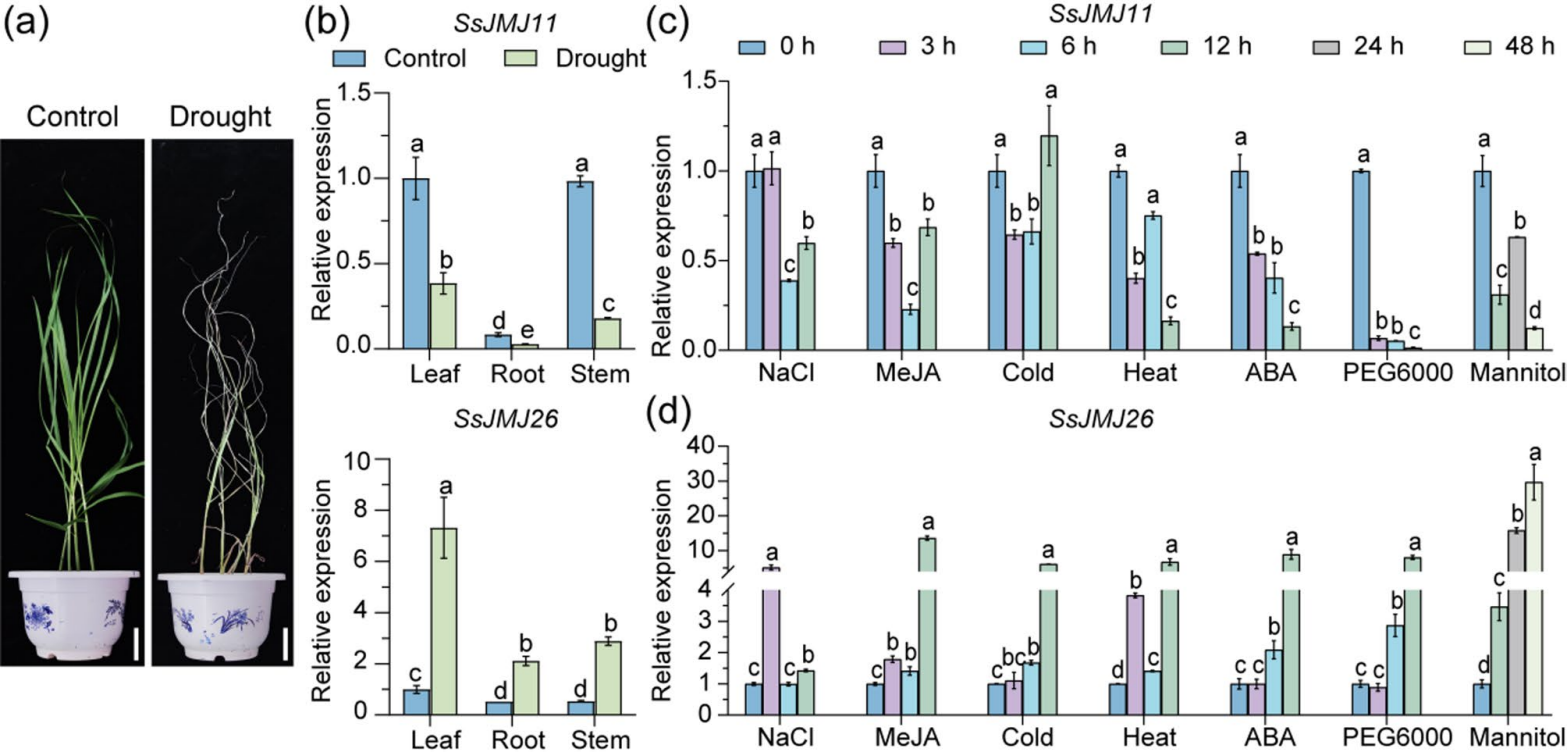
The expression of *SsJMJ11* and *SsJMJ26* genes in response to drought stress. (**a**) The phenotype of *S. spontaneum* watered normally or suffered from drought stress (RSWC, 10%). Bar = 5 cm. (**b**) RT-qPCR analysis of the *SsJMJ11* and *SsJMJ26* expression in leaf, root and stem of *S. spontaneum* in response to drought stress. (**c**, **d**) RT-qPCR analysis of the expression of *SsJMJ11* and *SsJMJ26* genes in response to NaCl, MeJA, Cold, Heat, ABA, PEG and Mannitol treatment. Four-week-old *S. spontaneum* seedlings were treated with 100 µM ABA, 200 mM NaCl, 100 mM Mannitol, 30% PEG, Cold (4℃), Heat (38℃), 100 µM MeJA for the indicated time. Both *SseEF* and *SsACTIN2* were used as internal controls. As similar results were obtained using different reference genes, only the results based on *SseEF* are presented. Data are presented as means ± SD (n = 3). Different letters denote significance difference between samples within each treatment (ANOVO with Turkey’ post hoc test, *P* < 0.05)

Our previous study revealed that SsJMJ11 and SsJMJ26 were H3K4 demethylases in *S. spontaneum* and their expression was regulated by drought stress [28, 29]. Therefore, we first evaluated the expression patterns of *SsJMJ11* and *SsJMJ26* in different tissues of *S. spontaneum* under drought stress conditions. Consistent with our previous report, the transcript level of *SsJMJ11* significantly declined in leaves, roots, and stems after drought stress, while the expression of *SsJMJ26* increased in all test tissues following drought stress (Fig. 1a and b).

Next, we investigated the responses of *SsJMJ11* and *SsJMJ26* in *S. spontaneum’s* seedlings to ABA, MeJA and NaCl, low temperature (4 °C), high temperature (38 °C), and osmotic stress (mannitol and PEG6000). The results showed that *SsJMJ11* was downregulated by various treatments, except for a slight upregulation observed in cold treatment for 12 h (Fig. 1c). In contrast, *SsJMJ26* was upregulated under all tested stress conditions (Fig. 1d). These results suggested that SsJMJ11 and SsJMJ26 may be involved in drought stress responses.

### Overexpression of *SsJMJ11* and *SsJMJ26* in Arabidopsis enhances sensitivity to drought stress

**Fig. 2:**
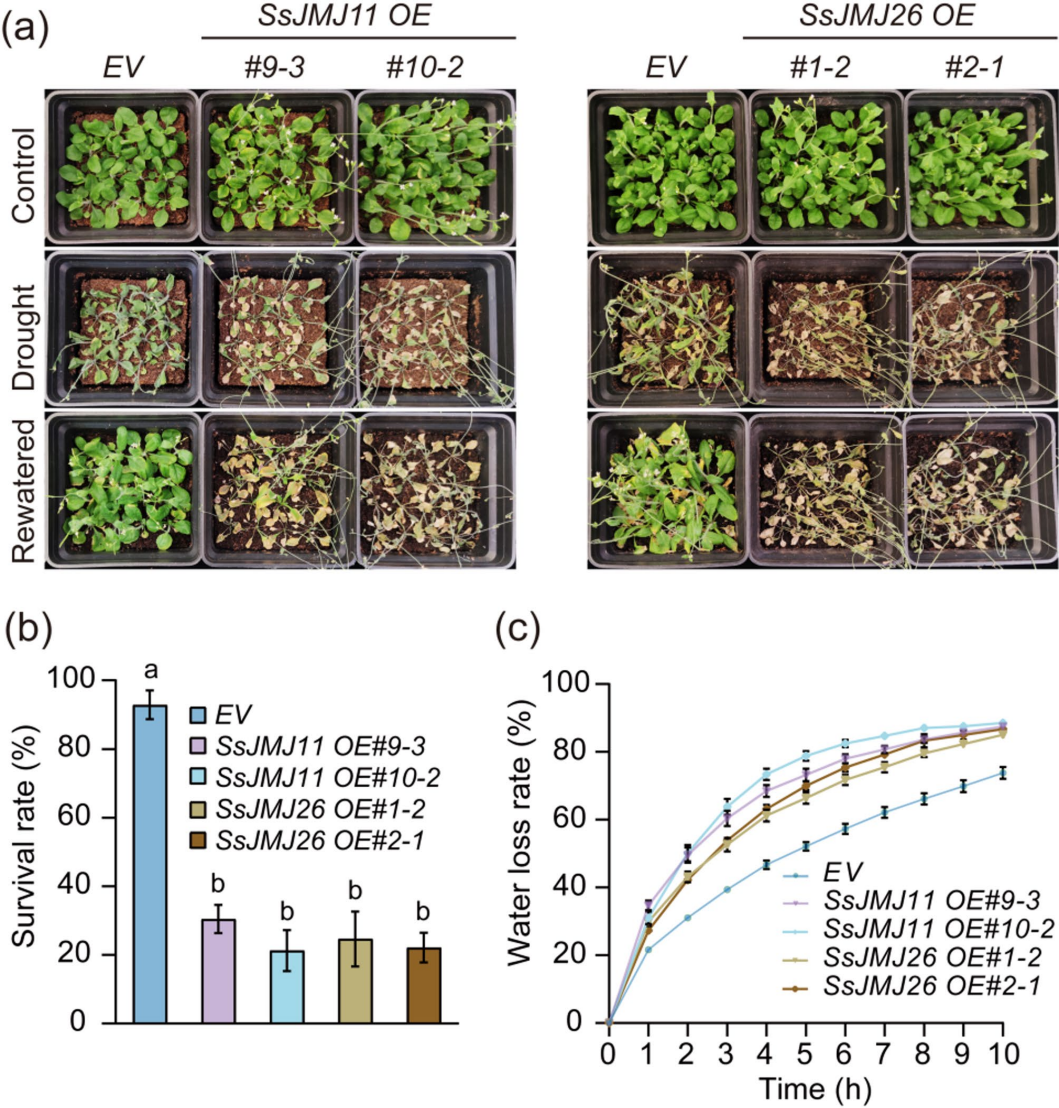
Overexpression of *SsJMJ11* and *SsJMJ26* in Arabidopsis enhance sensitivity to drought stress. (**a**) Drought sensitivity of *EV*, *SsJMJ11 OE* and *SsJMJ26 OE* transgenic Arabidopsis grown in soil. (**b**) Survival rates of *EV*, *SsJMJ11 OE* and *SsJMJ26 OE* plants after re-watering. Data represent means ± SD (n = 6). (**c**) Water loss rate of *EV*, *SsJMJ11 OE* and *SsJMJ26 OE* detached leaves. Data represent means ± SD (n = 4). All different letters represent statistically significant differences between them (ANOVO with Turkey’ post hoc test, *P* < 0.05)

To investigate whether SsJMJ11 and SsJMJ26 regulate drought tolerance, transgenic Arabidopsis lines overexpressing *SsJMJ11* (*SsJMJ11 OE*) and *SsJMJ26* (*SsJMJ26 OE*) were generated and subjected to soil drought stress. The *SsJMJ11 OE* and *SsJMJ26 OE* transgenic plants did not exhibit abnormal growth and developmental phenotypes except for a slight early flowering phenotype of *SsJMJ11 OE* under normal conditions [29]. The survival rates of both *SsJMJ11 OE* and *SsJMJ26 OE* transgenic plants were significantly lower than that of *EV* (empty vector OE) (Fig. 2a and b). In addition, significantly higher water loss rates from detached leaves of *SsJMJ11 OE* and *SsJMJ26 OE* plants were observed (Fig. 2c). These results suggest that overexpression of *SsJMJ11* and *SsJMJ26* enhance drought stress sensitivity in Arabidopsis, which might be caused by the increased water loss.

**Fig. 3:**
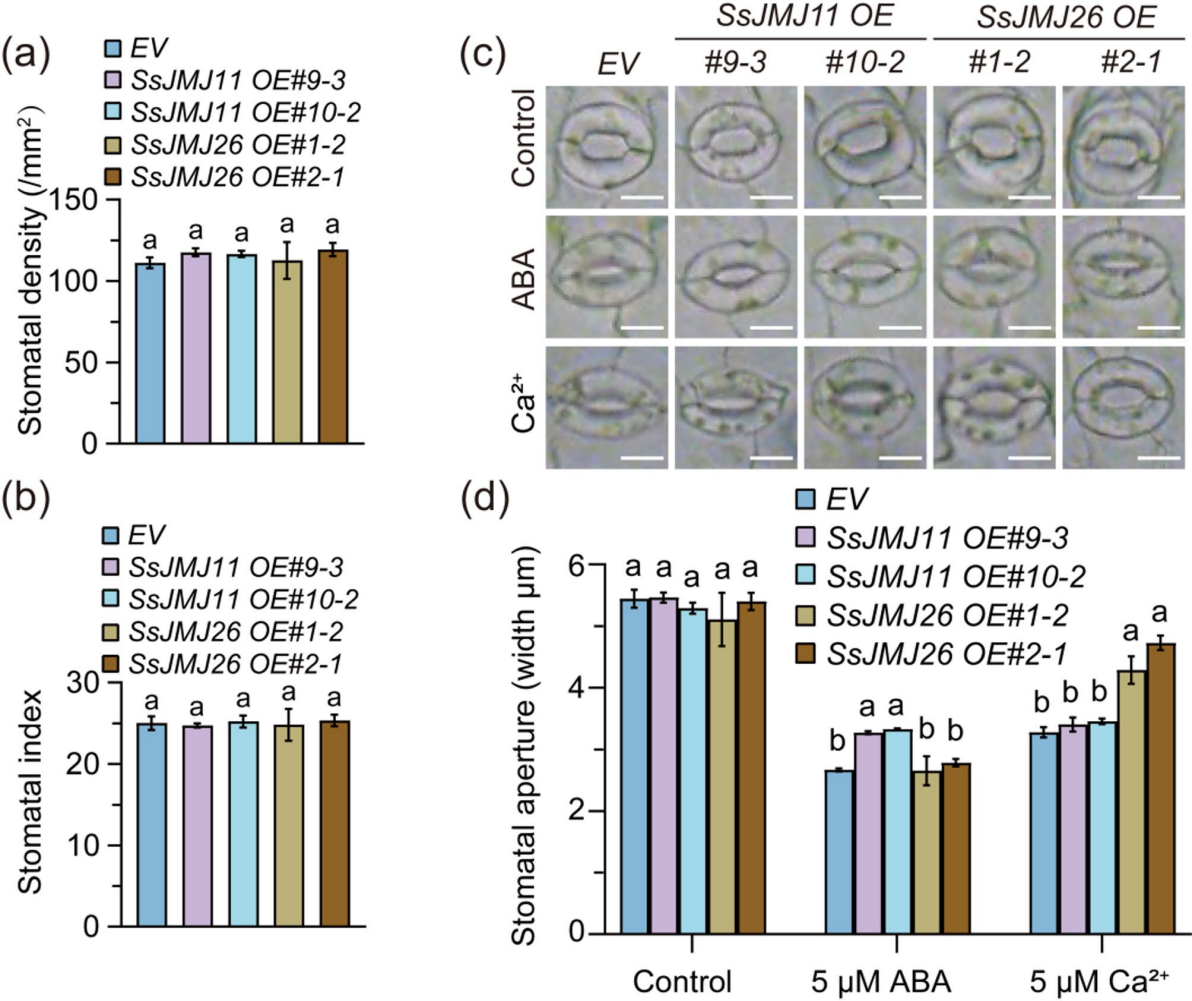
SsJMJ11 and SsJMJ26 negatively regulate stomatal closure. (**a**) Stomatal density and (**b**) Stomatal index in abaxial epidermis of leaves from three-week-old *EV*, *SsJMJ11 OE* and *SsJMJ26 OE* plants. Data shown as means ± SD (n = 10). (**c**) The stomatal aperture phenotype of *EV*, *SsJMJ11 OE* and *SsJMJ26 OE* plants in response to ABA and Ca^2+^. Bar, 10 µm. (**c**) Quantization of *EV*, *SsJMJ11 OE* and *SsJMJ26 OE* plants response to ABA- and Ca^2+^-induced stomatal closure. Data represent means ± SD (n = 60). All different letters represent statistically significant differences between genotypes within each treatment (ANOVO with Turkey’ post hoc test, *P* < 0.05)

Since there was a close relationship between water loss and stomatal regulation, *SsJMJ11* and *SsJMJ26* might influence drought tolerance through modulating stomata-related physiological processes. Therefore, we first conducted the stomatal density and index analysis to examine the effects of SsJMJ11 and SsJMJ26 on stomatal development. The results showed that overexpressing *SsJMJ11* and *SsJMJ26* did not significantly alter stomatal density or index in Arabidopsis (Fig. 3a and b). Next, stomatal movement assays were conducted to investigate the impacts of *SsJMJ11* and *SsJMJ26* on stomatal closure mediated by ABA and Ca^2+^, which are critical signals promoting stomatal closure under drought stress [33, 34]. Overexpression of *SsJMJ11* significantly inhibited ABA-mediated stomatal closure, but had no effect on Ca^2+^-mediated stomatal closure (Fig. 3c and d). In contrast, overexpression of *SsJMJ26* did not alter ABA-mediated stomatal closure but reduced Ca^2+^-mediated stomatal closure (Fig. 3c and d). These findings reveal the distinct roles of *SsJMJ11* and *SsJMJ26* in the ABA and Ca^2+^ signaling pathways, suggesting that they may regulate drought stress responses through different mechanisms.

### SsJMJ11 aggravates ABA-, NaCl- and mannitol-induced Inhibition of root growth and seed germination

**Fig. 4:**
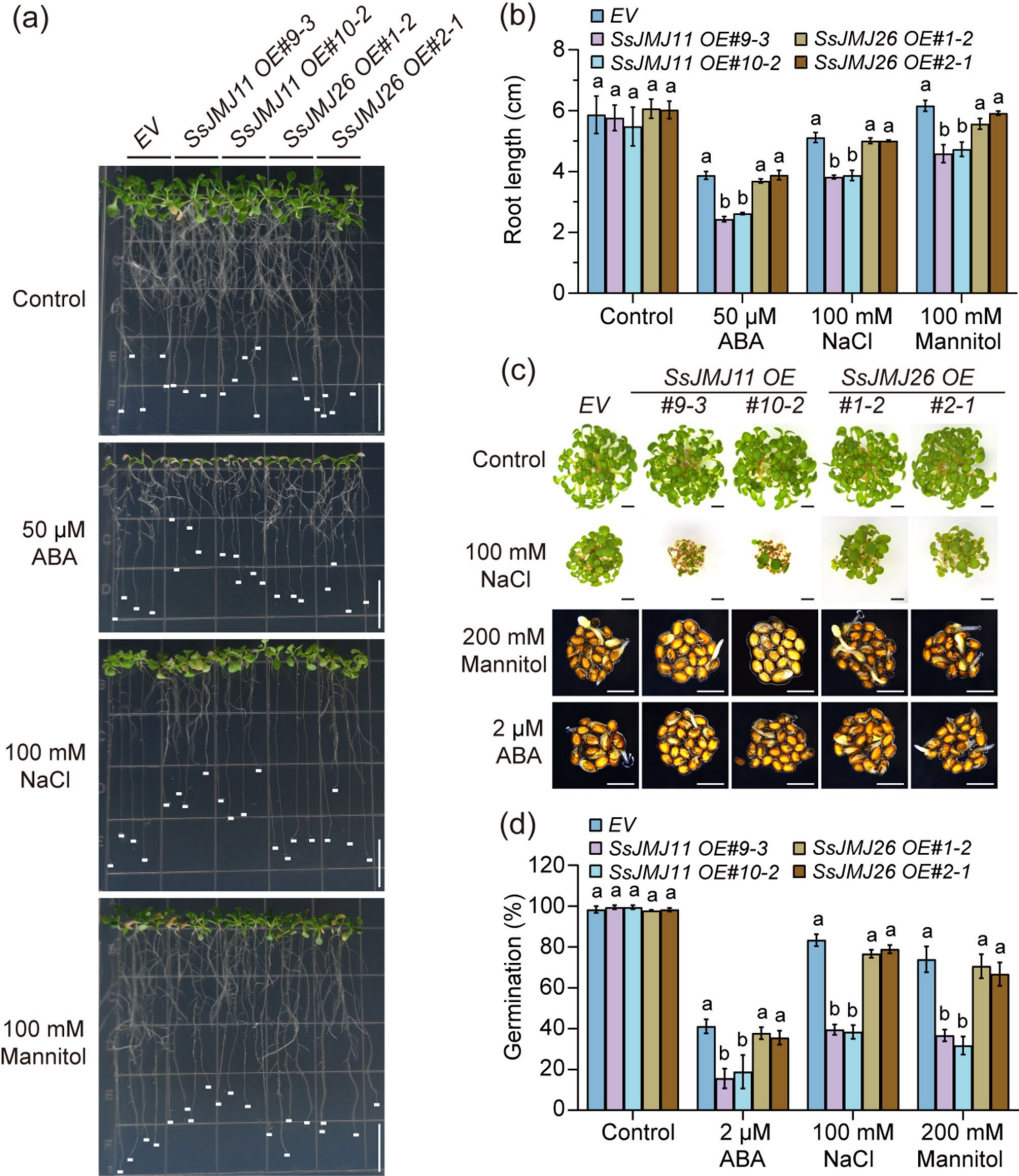
Overexpression of *SsJMJ11* and *SsJMJ26* in Arabidopsis enhanced the sensitivity to ABA, NaCl and Mannitol. (**a**) Photographs of root lengths of *EV*, *SsJMJ11 OE* and *SsJMJ26 OE* plants supplemented with ABA, NaCl and Mannitol. 5 d-old plants with similarly roots length of *EV*, *SsJMJ11 OE* and *SsJMJ26 OE* plants were transferred to 1/2 MS medium (Control), 1/2 MS medium containing 50 µM ABA, 100 mM NaCl and 100 mM Mannitol for growing another 7 d. Bars, 1 cm. (**b**) Measurements of root lengths of (a). Data represent means ± SD (n = 20). (c-d) Photographs of seed germination of *EV*, *SsJMJ11 OE* and *SsJMJ26 OE* seeds supplemented with ABA, NaCl and Mannitol. *EV*, *SsJMJ11 OE* and *SsJMJ26 OE* seeds germinated on 1/2 MS medium containing 2 µM ABA, 100 mM NaCl and 200 mM Mannitol for 10 days. (**d**) Quantization of seed germination of (c). Data represent means ± SD (n = 6). All different letters represent statistically significant differences between genotypes within each treatment (ANOVO with Turkey’ post hoc test, *P* < 0.05)

To further explore the role of *SsJMJ11* and *SsJMJ26* in stress responses, the effects of *SsJMJ11* and *SsJMJ26* overexpression on ABA-, NaCl-, and mannitol-mediated primary root growth and seed germination inhibition were analyzed. Under normal growth conditions, the primary root length was similar among *EV*, *SsJMJ11 OE* and *SsJMJ26 OE* lines (Fig. 4a and b). ABA and NaCl supplements inhibit the elongation of the primary roots of all tested plants and overexpression of *SsJMJ11*, but not *SsJMJ26*, significantly aggravated ABA-, and NaCl-mediated primary root growth inhibition (Fig. 4a and b). Although the primary root growth was not inhibited by mannitol treatment in the *EV* plants, but we still observed a shorter primary root length in *SsJMJ11 OE* plants but not in *SsJMJ26 OE* plants compared to that in *EV* plants (Fig. 4a and b). Meanwhile, overexpression of *SsJMJ11* and *SsJMJ26* had no influence on the seed germination under control conditions. However, overexpression of *SsJMJ11*, but not *SsJMJ26*, significantly aggravated ABA-, NaCl-, and mannitol-mediated seed germination inhibition. These results indicate that overexpression of *SsJMJ11*, but not *SsJMJ26* resulted in a hypersensitivity to ABA, NaCl, and mannitol, providing additional evidence for the involvement of SsJMJ11 in drought stress responses.

### SsJMJ11 suppresses the expression of *RD20* and *DREB2A* under dehydration stress

As H3K4me3 is associated with active gene expression, it is possible that SsJMJ11 negatively regulates drought-stress responses by repressing the expression of drought stress positive regulators. Thus, to gain insight into the molecular mechanisms of *SsJMJ11* function in responses to drought stress, we examined the expression of a number of positive regulators of ABA signaling and/or drought stress, including *AtABI5*,* AtOST1*,* AtABF1*,* AtABF2*,* AtABF3*, *AtABF4*, *AtDREB2A*,* AtGOLS2*,* AtRD20*,* AtERD1*,* AtERD10*,* AtERD11* and *AtHB7* [35–43]. All of the tested genes were induced by dehydration treatment (Fig. S1). Interestingly, among the tested genes, only the expression of *AtRD20* and *AtDREB2A* was greatly reduced in *SsJMJ11 OE* plants compared with *EV* plants under drought conditions (Fig. S1 and 5a). These results suggest that SsJMJ11 might negatively regulate Arabidopsis drought tolerance by reducing the transcription of *AtRD20* and *AtDREB2A*.

**Fig. 5:**
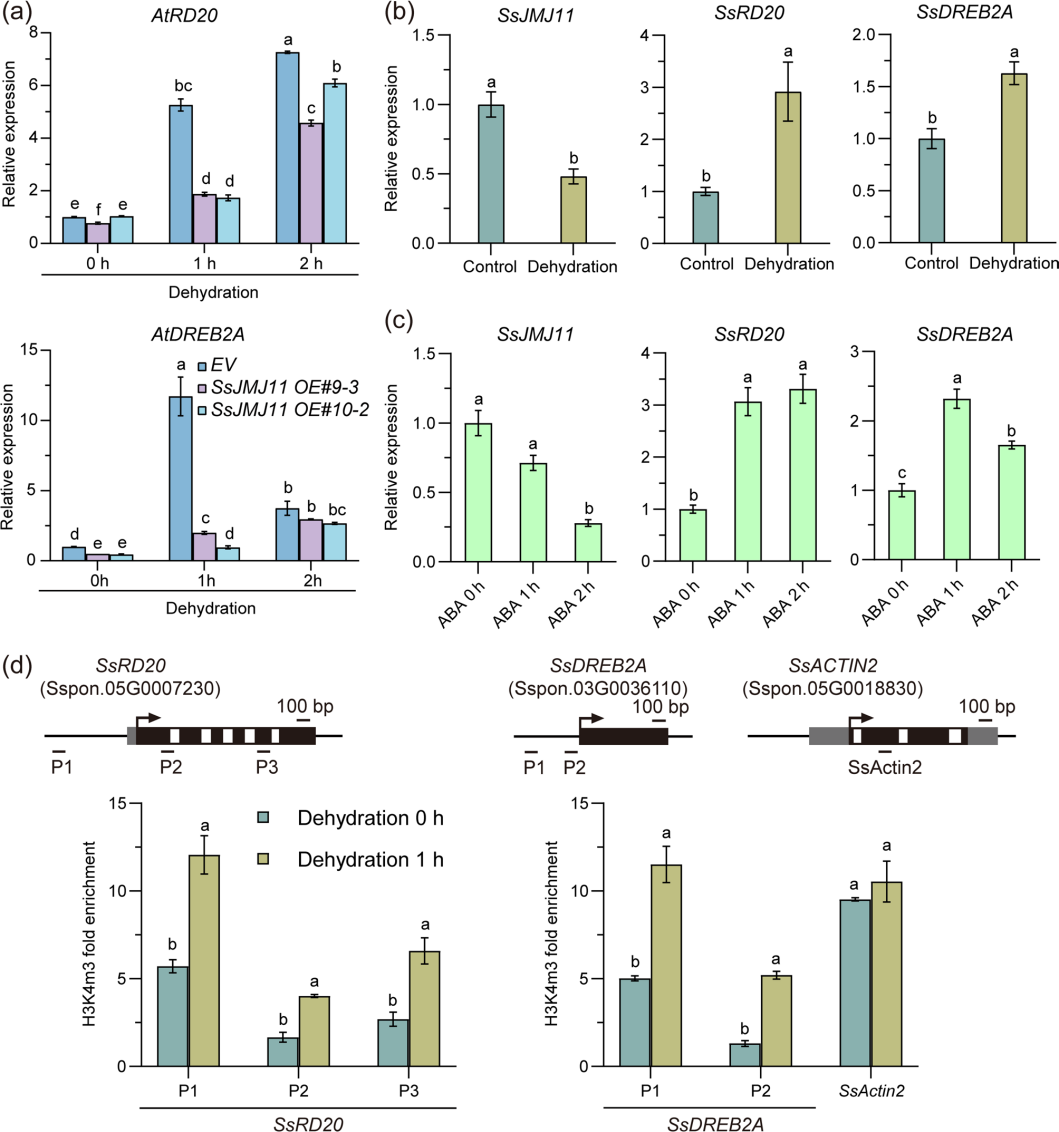
*SsRD20* and *SsDREB2A* induced by dehydration associated with the increased H3K4me3 levels in *S. spontaneum.* (**a**) RT-qPCR analysis of *AtRD20* and *AtDREB2A* genes in 3-week-old *EV* and *SsJMJ11* overexpression plants subjected to dehydration treatment for 0, 1, and 2 hours. RT-qPCR analysis of *SsJMJ11*,* SsRD20* and *SsDREB2A* genes in 1-month-old *S. spontaneum* seedlings subjected to dehydration treatment for 0, 1 hours (**b**) and 200 µM ABA treatment for 0, 1 and 2 hours (**c)**. (**d**) ChIP-qPCR analysis of H3K4me3 levels at the *SsRD20* and *SsDREB2A* loci of *S. spontaneum* seedlings in response to dehydration treatment for 0, 1 hours. Both *AtUBC/AtACTIN2* and *SseEF/SsACTIN2* were used as the internal controls. As similar results were obtained using different reference genes, only the results based on *AtUBC/SseEF* are presented. Data are presented as means ± SD (n = 3). Different letters denote statistically significant differences as determined by ANOVO with Turkey’ post hoc test (*P* < 0.05)

In order to further confirm the role of SsJMJ11 in the regulation of drought-stress responses in *S. spontaneum*, we analyzed the expression and H3K4me3 deposition of *SsRD20* (Sspon.05G0007230) and *SsDREB2A* (Sspon.03G0036110), the orthologous genes of *AtRD20* and *AtDREB2A*, respectively, in *S. spontaneum* seedlings under drought stress conditions (Fig. S2). The results revealed that dehydration and ABA treatments promoted the expression of *SsRD20* and *SsDREB2A* (Fig. 5b and c). Besides, the ChIP-qPCR analysis showed that the induction of *SsRD20* and *SsDREB2A* expression was concomitant with an increase in H3K4me3 levels in response to dehydration stress (Fig. 5d). These results suggest that SsJMJ11 might also negatively regulate drought tolerance by reducing the transcription of *SsRD20* and *SsDREB2A* in *S. spontaneum*.

### SsJMJ11 inhibits the expression of *SsRD20* and *SsDREB2A* by suppressing the H3K4me3 in *S. spontaneum*

**Fig. 6:**
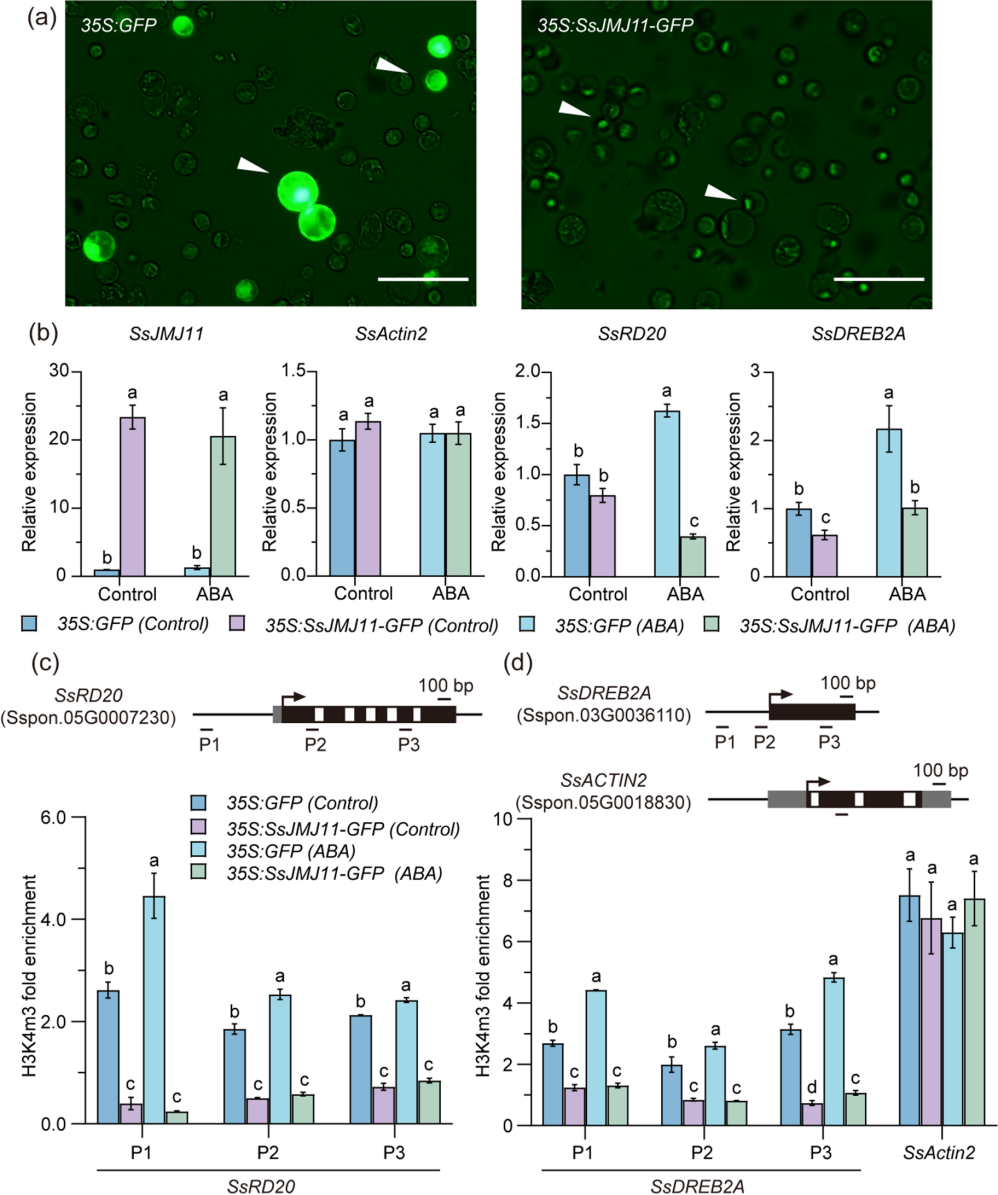
SsJMJ11 reduces the expression of *SsRD20*,* SsDREB2A* through decreasing the H3K4me3 modification levels in *S. spontaneum* protoplasts. (**a**) Transient expression of *35S: GFP* and *35S: SsJMJ11-GFP* in *S. spontaneum* protoplasts. White arrows indicate protoplasts expressing GFP or SsJMJ11-GFP. Scale bar = 50 µm. (**b**) RT-qPCR analysis of *SsJMJ11*,* SsRD20*,* SsDREB2A*, and *SsACTIN2* expression in *S. spontaneum* protoplasts expressing GFP or SsJMJ11-GFP. The control group did not receive ABA treatment, whereas the ABA group received 200 µM ABA treatment for 2 h. *SseEF* served as an internal control. Data are presented as means ± SD (n = 3). Significant differences (ANOVO with Turkey’ post hoc test, *P* < 0.05) are indicated by different letters. (**c**) ChIP-qPCR analysis was performed to assess H3K4me3 levels at the *SsRD20*,* SsDREB2A*, and *SsACTIN2* loci in *S. spontaneum* protoplasts transfected with either GFP or SsJMJ11-GFP. The data presented are means ± standard deviation from three independent experiments (n = 3). Different letters denote statistically significant differences between groups as determined by ANOVO with Turkey’ post hoc test (*P* < 0.05)

Furthermore, to confirm the potential association between *SsJMJ11* and *SsRD20*, *SsDREB2A* in *S. spontaneum*, we performed transient transformation system to express *SsJMJ11-GFP* in *S. spontaneum* leaf protoplasts (Fig. 6a). The transcript levels of *SsJMJ11* increased approximately 24.5-fold in *SsJMJ11-GFP* transformed protoplasts compared to those in *GFP* transformed protoplasts (Fig. 6b). Moreover, RT-qPCR analysis revealed that ABA treatment significantly promoted the expression of *SsRD20* and *SsDREB2A* in *GFP* transformed protoplasts. However, the ABA-elevated expression of *SsRD20* and *SsDREB2A* was remarkably inhibited by overexpression of *SsJMJ11-GFP* (Fig. 6b). Furthermore, ChIP-qPCR analysis with a specific anti-H3K4me3 antibody revealed that ABA treatment increased H3K4me3 levels at the *SsRD20* and *SsDREB2A* loci in *GFP* transformed protoplasts, but not in SsJMJ11-GFP expressed protoplasts (Fig. 6c and d). Overexpression of *SsJMJ11-GFP* remarkably reduced H3K4me3 levels at the *SsRD20* and *SsDREB2A* loci under normal conditions and abolished the ABA-induced increase of H3K4me3 levels of *SsRD20* and *SsDREB2A* loci. These results suggest that SsJMJ11 negatively regulates the expression of *SsRD20* and *SsDREB2A* by removing their H3K4me3 modification in *S. spontaneum*.

## Discussion

Drought stress severely limits sugarcane production for almost all sugarcane-producing countries due to global warming. However, the sugarcane genome is among the most complex of cultivated crops. Although stable genetic transformation systems and CRISPR/Cas-mediated genome editing in sugarcane have been reported, the large genome size, polyploidy, low transformation efficiency, recalcitrance to genetic transformation, difficulty in vitro regeneration, and the long lifespan have always imposed a challenge for the functional characterization of genes [4, 30]. Recently, we developed an efficient transient transformation system based on *S. spontaneum* protoplasts for homologous functional analysis of sugarcane genes [30]. Based on this transformation system, we demonstrated that the sugarcane H3K27 demethylase SsJMJ4 negatively regulates drought-stress responses by removing H3K27me3 from *SsWRKY122* loci and thereby promoting its expression [28]. Meanwhile, the H3K4me3 demethylase SsJMJ11 promotes sugarcane flowering by removing H3K4me3 from *SsCDFs* loci and repressing their expression [29]. In the present study, the role of SsJMJ11 was characterized through both ectopic overexpression of *SsJMJ11* in Arabidopsis and homologous overexpression in *S. spontaneum* protoplasts. Our findings suggest that SsJMJ11 negatively regulates drought tolerance by removing H3K4me3 from *SsRD20* and *SsDREB2A* loci, and that SsJMJ11-mediated dynamic deposition of H3K4me3 is involved in drought stress responses. Future work on functional validation of this module in stably transformed sugarcane plants and CRISPR/Cas-mediated loss-of-function sugarcane mutants will provide better understanding of drought tolerance for sugarcane improvement.

Under drought conditions, the expression of drought-responsive genes is generally associated with substantial changes in histone modifications [7, 44]. Histone demethylase-mediated histone demethylation is essential for the dynamic alteration of dehydration-responsive genes, and several histone demethylases have been shown to function in response to drought stress through their specific downstream stress-responsive genes. In Arabidopsis, the H3K4me3 demethylases AtJMJ17 negatively regulates dehydration tolerance and ABA response by decreasing the transcription of *AtOST1* and *AtABI5*, respectively [25, 45]. In rice, the H3K4me3 demethylase OsJMJ703 downregulates abundant dehydration genes under drought stress [27]. The H3K36 demethylase OsJMJ710 negatively regulates drought stress by suppressing *OsMYB48-1* expression [46]. In soybean, H3K27 demethylases GmJMJ30 have been shown to enhance drought tolerance by increasing *GmZF351* expression [47]. In sugarcane, we recently reported that H3K27 demethylases SsJMJ4 negatively regulates drought-stress by raising the transcription of *SsWRKY122* mediated by histone demethylation [28]. In this study, we found that the Jumonji domain-containing H3K4me3 demethylase SsJMJ11 and SsJMJ26 negatively regulate drought-stress responses in *A.thaliana*. Ectopic overexpression of *SsJMJ11* and *SsJMJ26* in Arabidopsis displayed drought-stress-sensitive phenotypes (Fig. 2). Moreover, SsJMJ11 overexpression lines were insensitive to ABA-mediated stomatal closure (Fig. 3). Further results suggested that SsJMJ11 downregulated the expression of drought-positive regulators *AtRD20* and *AtDREB2A* under drought stress (Fig. 5a). Similarly, *SsRD20* and *SsDREB2A* expression levels were induced by ABA and dehydration treatment and were associated with increased H3K4me3 levels at their loci (Fig. 5b and d). Furthermore, we also confirmed that H3K4me3 methylation levels of *SsRD20* and *SsDREB2A* loci were dramatically reduced, which was associated with decreased transcription of these genes when SsJMJ11 was overexpressed in *S. spontaneum* protoplasts (Fig. 6b). Moreover, SsJMJ11 impaired ABA-induced transcription and H3K4me3 levels of *SsRD20* and *SsDREB2A* loci (Fig. 6c and d). Both AtRD20 and AtDREB2A are well-known ABA-responsive positive regulators of drought tolerance. AtRD20 positively regulates ABA-mediated stomatal closure, as well as inhibition of germination, and hence drought responses [43]. AtDREB2A enhances drought stress tolerance by activating drought stress-responsive gene expression [48, 49]. Therefore, these results suggest that SsJMJ11-mediated regulation of H3K4 methylation status is associated with transcriptional regulation of drought-stress genes, and hence negatively regulates drought tolerance.

Similarly, the Arabidopsis H3K4me3 demethylase AtJMJ17 and the rice H3K4me3 demethylase OsJMJ703 have been found to negatively regulate dehydration tolerance and/or ABA response by regulating the H3K4 methylation status of dehydration genes under drought stress [25, 27, 45], suggesting that the regulatory role of H3K4 methylation in drought stress response appears to be conserved across different plant species. Moreover, AtJMJ14 was reported to be recruited to its specific target sites by AtNAC050 and AtNAC052 transcription factors in Arabidopsis [50]. And AtJMJ17 could be recruited by the AtWRKY40 transcription factor to *AtABI5* chromatin and regulates drought stress responses in Arabidopsis [25, 45]. It is reasonable to hypothesize that SsJMJ11 might be directed to its target sites by a transcription factor(s) with DNA binding activity, which need to be identified in future studies. In addition, the induction of *AtRD20* by dehydration was also observed in *SsJMJ11* overexpressed lines. This suggests that there might exist other factors together with SsJMJ11 regulating the expression of *AtRD20*. Our previous study indicated that drought induced the accumulation of AtJMJ27, hence promoting the expression of *AtRD20* by reducing the H3K9me2 levels at its chromatin loci [26]. Therefore, it is possible that there might exist other factors, such as AtJMJ27, regulating the induction of *AtRD20* by drought, which need to be explored in future studies.

There are 7 non-redundant KDM5 subfamily JmjC domain-containing proteins in *S. spontaneum.* However, only SsJMJ11 and SsJMJ26 contain intact conserved residues for demethylase activity [28]. Although both SsJMJ26 and SsJMJ11 possess H3K4me3 demethylase activity, their roles in drought responses are distinct. First, the expression of *SsJMJ11* was depressed by drought, whereas that of *SsJMJ26* was upregulated. Second, SsJMJ11 affects ABA-mediated stomatal closure, while SsJMJ26 reduces Ca^2+^-mediated stomatal closure. Thirdly, SsJMJ11 aggravates ABA-, NaCl-, and mannitol-induced inhibition of root growth and seed germination, whereas SsJMJ26 has no influence on them. Since Ca^2+^-mediated stomatal closure is known to involve both ABA-dependent and ABA-independent pathways [51], it seems conceivable that SsJMJ11 and SsJMJ26 might function through ABA-dependent and ABA-independent signaling, respectively, to negatively regulate drought stress in *A. thaliana*. However, the molecular mechanism of SsJMJ26 in drought response regulation remains to be further determined. Similarly, H3K27 demethylase ELF6 (AtJMJ11) delays flowering by acting as a suppressor of the photoperiod pathway [52], while REF6 (AtJMJ12), which is most homologous to AtJMJ11, promotes flowering as an AtFLC repressor [53]. This might be caused by their differential targeting. SsJMJ11 and SsJMJ26 might be directed to their specific target sites by different transcription factors and/or chromatin-associated proteins.

Additionally, H3K4me3, a core epigenetic mark for gene activation, plays a pivotal role in the establishment of plant stress memory and its transgenerational inheritance [54–58]. Our study reveals that SsJMJ11 specifically removes H3K4me3 modifications at key drought-responsive genes loci (e.g., *SsRD20* and *SsDREB2A*), thereby suppressing their transcriptional activity (Figs. 5d and 6c). It would be of interest to determine whether and how SsJMJ11 is involved in drought stress memory and its transgenerational inheritance in *S. spontaneum* in future studies.

## Conclusions

We characterized the novel function of the sugarcane H3K4me3 demethylase SsJMJ11 in regulating drought-stress responses, and SsJMJ11-mediated dynamic deposition of H3K4me3 is involved in the appropriate response to drought stress. We propose that SsJMJ11 might prevent silencing of positive regulators of drought stress to ensure proper growth under favorable conditions, while down-regulation of SsJMJ11 under drought stress contributes to the activation of drought-stress responses.

## Electronic supplementary material

Below is the link to the electronic supplementary material.

Supplementary Material 1

Supplementary Material 2

## Data Availability

Data is provided within the manuscript or supplementary information files.

## Abbreviations

ABA: Abscisic Acid
H3K4me3: Histone H3 Lysine 4 Trimethylation
TSS: Transcription Start Sites
JmjC: Jumonji C Domain
OST1: OPEN STOMATA 1
RSWC: Relative Soil Water Content
MeJA: Methyl Jasmonate
NaCl: Diverse Abiotic Stresses, Including Salt
RT-qPCR: Real-Time Quantitative Polymerase Chain Reaction
EV: Empty Vector OE
