# Supplementary material for: H3K4 demethylase SsJMJ11 negatively regulates drought-tolerance responses in sugarcane
Source: BMC Plant Biol. 2025 Jul 2;25:814. doi: 10.1186/s12870-025-06832-z (PMC12220489; doi:10.1186/s12870-025-06832-z)
Supplement: Supplementary file 1 — Supplementary Material 1 [file 12870_2025_6832_MOESM1_ESM.docx]

Supplemental table 1 qPCR primer sequences used for this research

| Name | DNA sequence (5' to 3') |  |
| --- | --- | --- |
| **Primers for RT-qPCR** | | |
| *SsJMJ11-qPCR-F* | TCGAGTGAGGACTCCGAAGA |  |
| *SsJMJ11-qPCR-R* | CGCCCTCAATTTCCTCCACT |  |
| *SsJMJ26-qPCR-F* | ACTGCTTCAAGAACACAAAA |  |
| *SsJMJ26-qPCR-R* | CATTTAGTTGCTGATTACCA |  |
| *SsRD20-qPCR-F* | GTCGTTCTTCGTCGCTGTTG |  |
| *SsRD20-qPCR-R* | CTGCCATGCTTGCTCTTGTG |  |
| *SsDREB2A-qPCR-F* | TTCAATCTGAGAAGAGTGTG |  |
| *SsDREB2A-qPCR-R* | ATTTTTTTATCAGCATTCAA |  |
| *SsACTIN2-qPCR-F* | TTGGACTCTGGTGATGGTGT |  |
| *SsACTIN2-qPCR-R* | GTGGTGGTGAAGGAGTAACC |  |
| *SseEF-qPCR-F* | TTTCACACTTGGAGTGAAGCAGAT |  |
| *SseEF-qPCR-R* | GACTTCCTTCACAATCTCATCATAA |  |
| *AtRD20-qPCR-F* | TTAGCTCCGGTCACCAGTCA |  |
| *AtRD20-qPCR-R* | CATGTATGGTTTTGGTAATGTTTCC |  |
| *AtDREB2A-qPCR-F* | GACCTAAATGGCGACGATGT |  |
| *AtDREB2A-qPCR-R* | TCGAGCTGAAACGGAGGTAT |  |
| *AtUBC-qPCR-F* | TTCAAATGGACCGCTCTTATC |  |
| *AtUBC-qPCR-R* | GCTCAGGATGAGCCATCAAT |  |
| *AtGOLS2-qPCR-F* | AAGTTGTTCACTACTGTGCT |  |
| *AtGOLS2-qPCR-R* | AGCTTCGATAAACTGCTGAA |  |
| *AtERD1-qPCR-F* | GCAGCAGGCGACGATGA |  |
| *AtERD1-qPCR-R* | TGCAACGGCTGCAATATCA |  |
| *AtERD10-qPCR-F* | TCTCTGAACCAGAGTCGTTT |  |
| *AtERD10-qPCR-R* | CTTCTTCTCACCGTCTTCAC |  |
| *AtERD11-qPCR-F* | AAAAGTGGCGATGACCTAAT |  |
| *AtERD11-qPCR-R* | ATTCAAATCAAACACTCGGC |  |
| *AtERF6-qPCR-F* | GAGAAAGTGCTAAAGACGGA |  |
| *AtERF6-qPCR-R* | TCAAACAACGGTCAATTGTG |  |
| *AtHB-7-qPCR-F* | CGAGGCAAGTGGCTATATGGTT |  |
| *AtHB-7-qPCR-R* | CGAGCTGCTTGGATTTCCA |  |
| *AtOST1-qPCR-F* | TAGCCATTGTTATGGAATATGCATC |  |
| *AtOST1-qPCR-R* | AACCTCGCCTCGTCTTCGCT |  |
| *AtABI5-qPCR-F* | GAGAATGCGCAGCTAAAACA |  |
| *AtABI5-qPCR-R* | GTGGACAACTCGGGTTCCTC |  |
| *AtABF1-qPCR-F* | TCAACAACTTAGGCGGCGATAC |  |
| *AtABF1-qPCR-R* | GCAACCGAAGATGTAGTAGTCA |  |
| *AtABF2-qPCR-F* | TTGGGGAATGAGCCACCAGGAG |  |
| *AtABF2-qPCR-R* | GACCCAAAATCTTTCCCTACAC |  |
| *AtABF3-qPCR-F* | CCGCAGAGGCAACAAACG |  |
| *AtABF3-qPCR-R* | CCAGCCCTGACCAAAAACTC |  |
| *AtABF4-qPCR-F* | AACAACTTAGGAGGTGGTGGTC |  |
| *AtABF4-qPCR-R* | CTTCAGGAGTTCATCCATGTTC |  |
|  |  |  |
| **Primers for ChIP-qPCR** | | |
| *SsRD20-P1-F* | CACACACGGCGACTGACGA |  |
| *SsRD20-P1-R* | AGTTCGCCCGTTGCTGCTA |  |
| *SsRD20-P2-F* | CGAAGGACTCCCAGACCAT |  |
| *SsRD20-P2-R* | ATGGCTTAGGGACGTGCTC |  |
| *SsRD20-P3-F* | TGTTCAGCAAGTACGCCCG |  |
| *SsRD20-P3-R* | AAGACTTCGCGGAACCCCT |  |
| *SsDREB2A-P1-F* | TGTCCTCTGGTTCTCCTTC |  |
| *SsDREB2A-P1-R* | ACAGAACAGAAGCACATCG |  |
| *SsDREB2A-P2-F* | GGAGGTCTGTTGTGTGGTT |  |
| *SsDREB2A-P2-R* | GAGTCGTTTTAGATGCTGT |  |
| *SsDREB2A-P3-F* | TGCTGGCATCTAGTGTACC |  |
| *SsDREB2A-P3-R* | TCATCATTCCCTTCTGTTT |  |
| *SsACTIN2-F* | GGCATCACACCTTCTATAATGA |  |
| *SsACTIN2-R* | CTCAAACATGATTTGGGTCA |  |

**
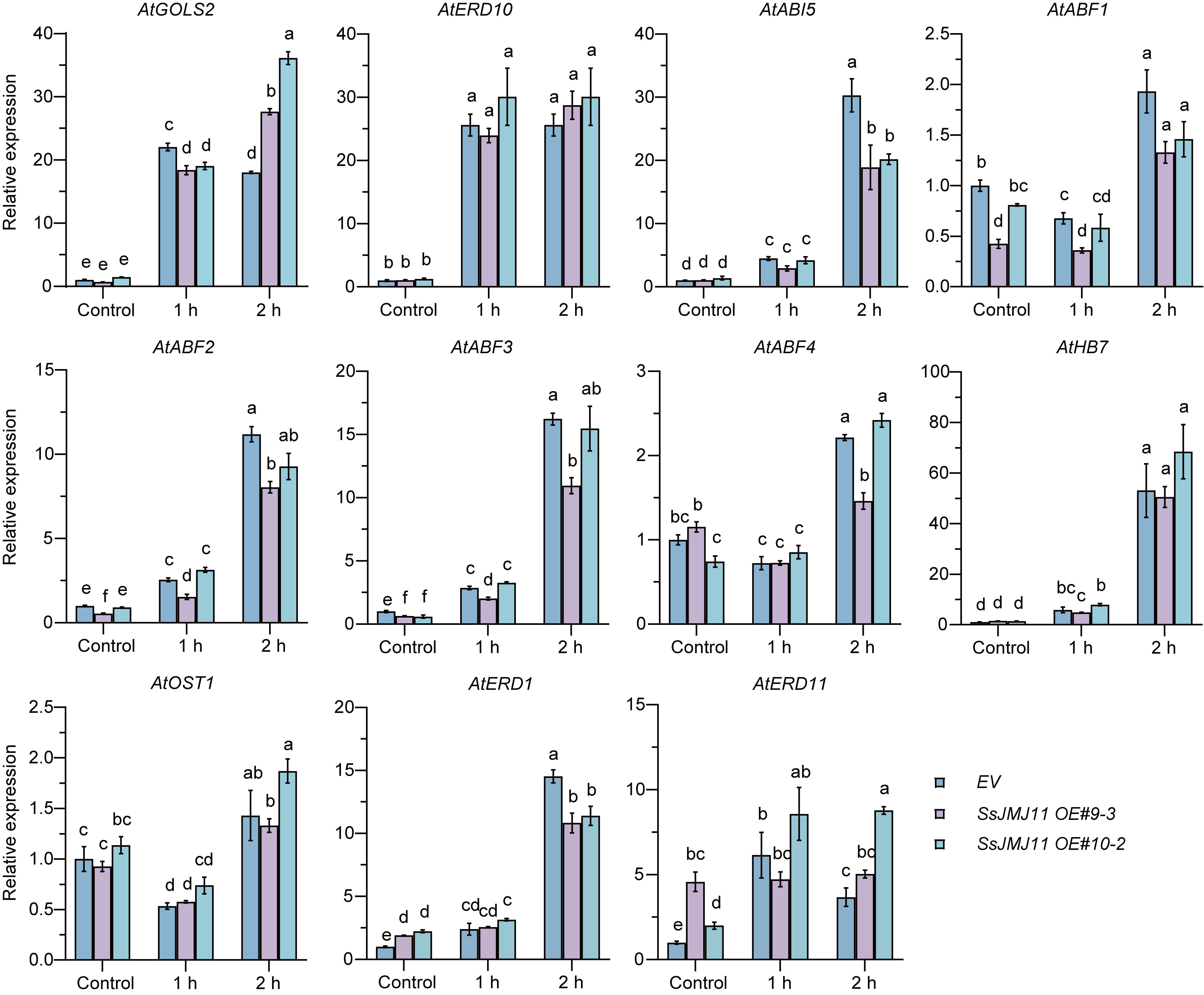
**

**Figure S1.** RT-qPCR analysis of stress responsive genes in response to dehydration stress**.** 3-week-old *EV* and *SsJMJ11* overexpression plants were exposed to dehydration treatment for 0, 1, and 2 hours. Both *AtUBC* and *AtActin2* were used as the internal controls. As similar results were obtained using different reference genes, only the results based on *AtUBC* are presented. Data are presented as means ± SD (n = 3). Different letters denote statistically significant differences as determined by ANOVO with Turkey’ post hoc test (P < 0.05).

**
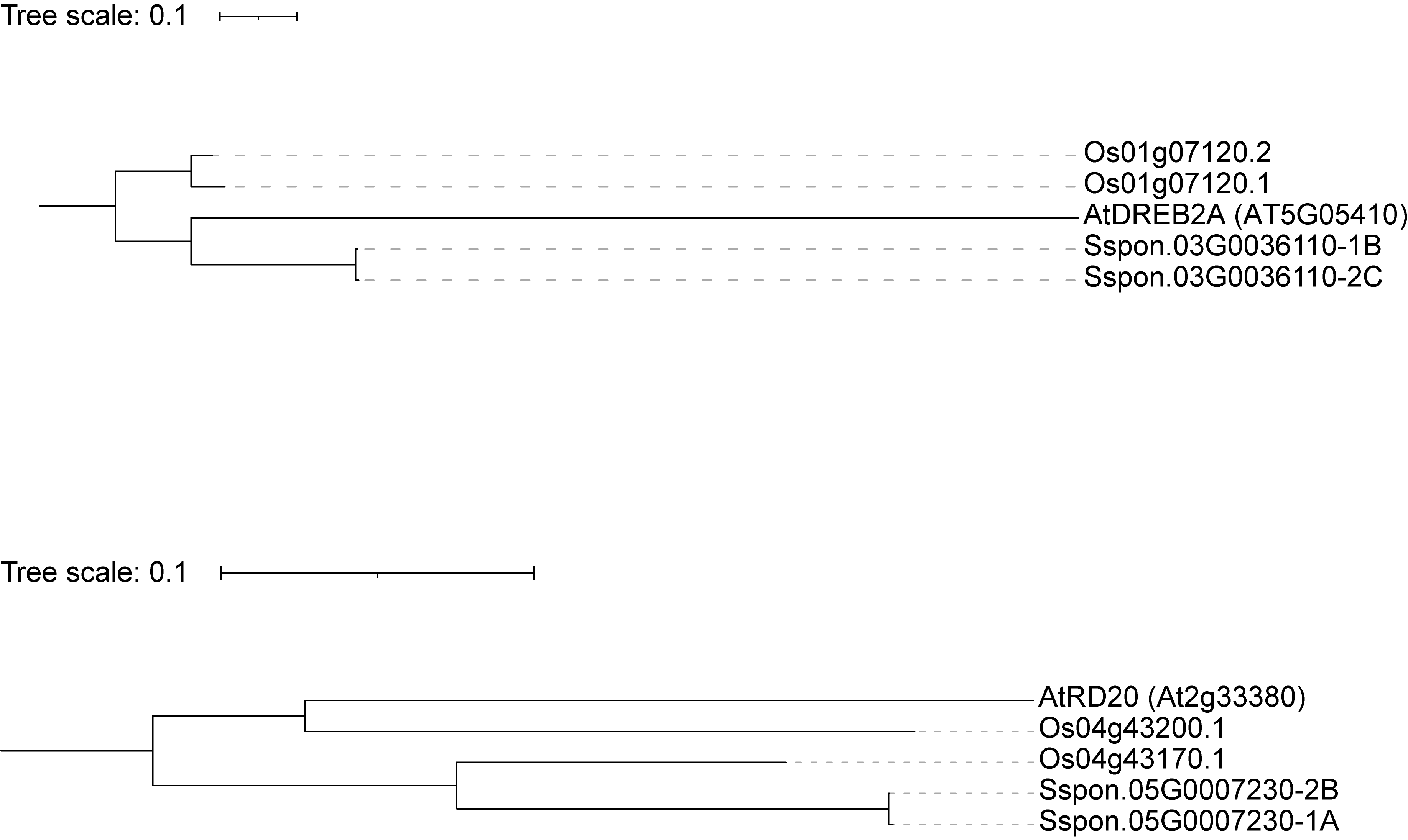
**

**Figure S2. OrthoFinder identified orthogroups of *RD20* and *DREB2A* across *A. thaliana*, *O. sativa*, and *S. spontaneum.***
