# Supplementary material for: H3K4 demethylase SsJMJ11 negatively regulates drought-tolerance responses in sugarcane
Source: BMC Plant Biol. 2025 Jul 2;25:814. doi: 10.1186/s12870-025-06832-z (PMC12220489; doi:10.1186/s12870-025-06832-z)
Supplement: Supplementary file 2 — Supplementary Material 2 [file 12870_2025_6832_MOESM2_ESM.docx]

**
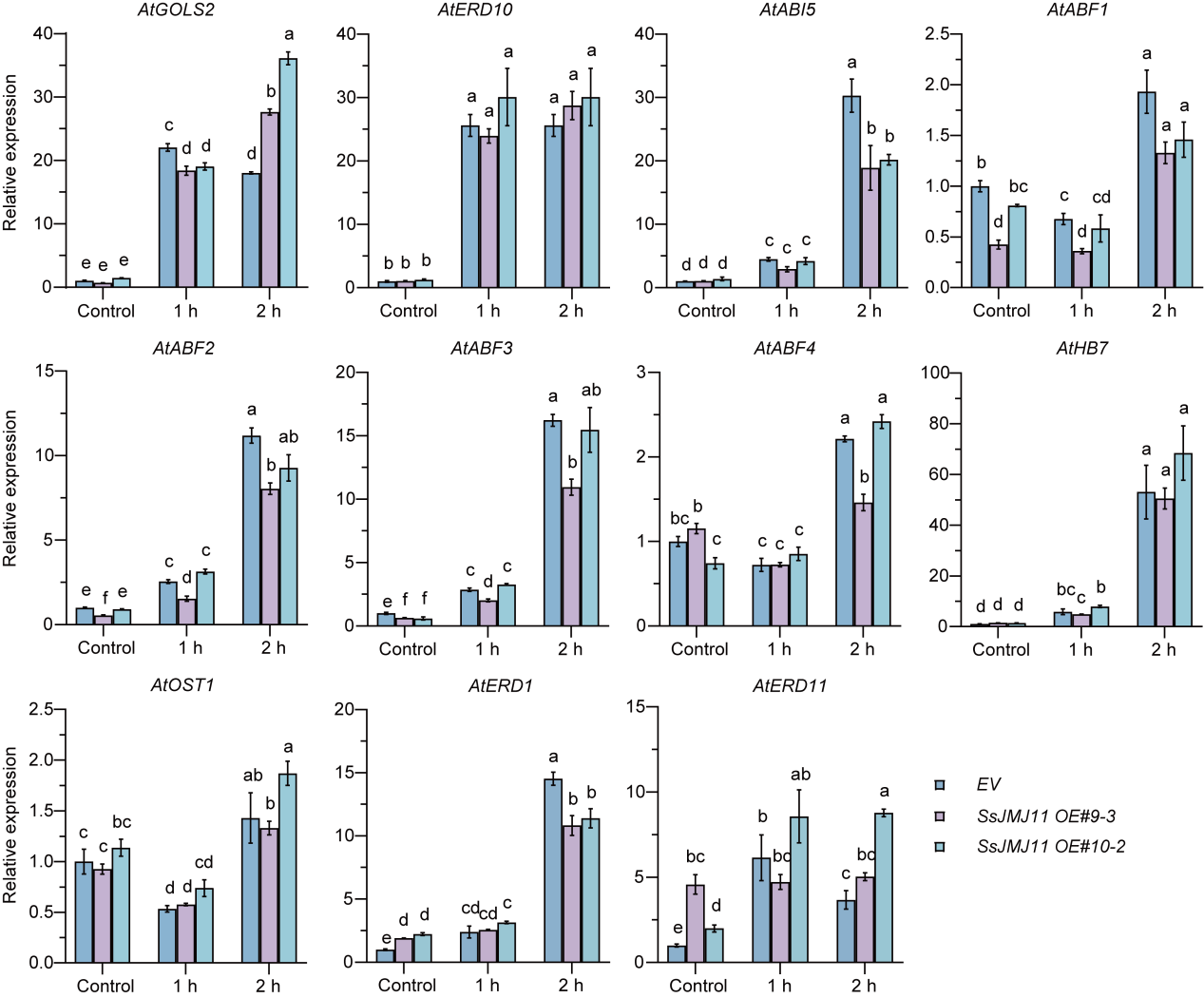
**

**Figure S1. RT-qPCR analysis of stress responsive genes in response to dehydration stress.**

3-week-old *EV* and *SsJMJ11* overexpression plants were exposed to dehydration treatment for 0, 1, and 2 hours. Both *AtUBC* and *AtActin2* were used as the internal controls. As similar results were obtained using different reference genes, only the results based on *AtUBC* are presented. Data are presented as means ± SD (n=3). Different letters denote statistically significant differences as determined by ANOVO with Turkey’ post hoc test (P<0.05).

**
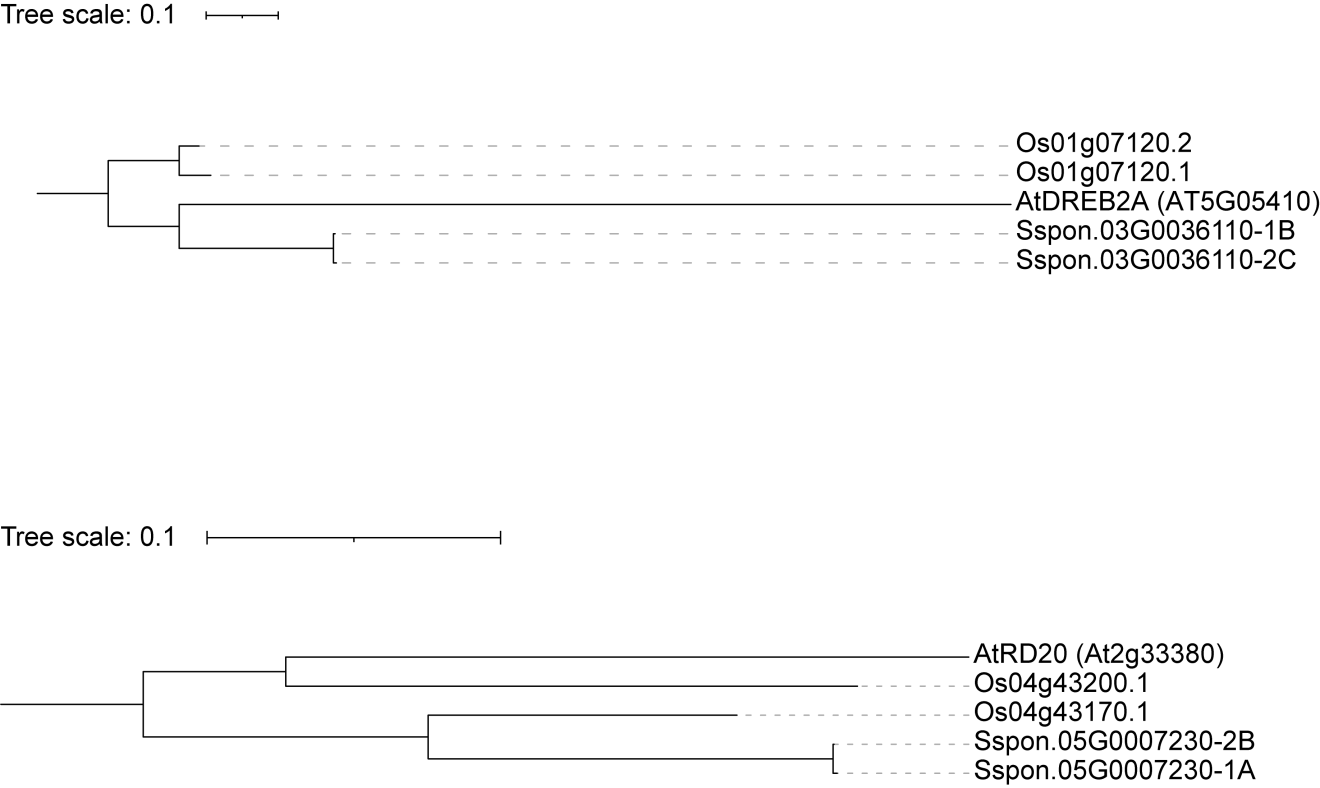
**

**Figure S2. OrthoFinder identified orthogroups of *RD20* and *DREB2A* across *A. thaliana*, *O. sativa*, and *S. spontaneum.***
